# Clinical Utility of Definitive Drug–Drug Interaction Testing in Primary Care

**DOI:** 10.3390/jcm7110384

**Published:** 2018-10-25

**Authors:** John Peabody, Mary Tran, David Paculdo, Joshua Schrecker, Czarlota Valdenor, Elaine Jeter

**Affiliations:** 1 Department of Epidemiology and Biostatistics/Department of Medicine, University of California, San Francisco, CA 94158, USA; 2 School of Public Health, University of California, Los Angeles, CA 90095, USA; 3 QURE Healthcare, San Francisco, CA 94133, USA; mtran@qurehealthcare.com (M.T.); dpaculdo@qurehealthcare.com (D.P.); czarlie@qurehealthcare.com (C.V.); 4 Aegis Sciences Corporation, Nashville, TN 37228, USA; joshua.schrecker@aegislabs.com (J.S.); elaine.jeter@aegislabs.com (E.J.)

**Keywords:** drug–drug interaction, drug–food interaction, drug–supplement interaction, medication reconciliation, adverse drug event, primary care, psychiatric medications, CNS depressants, opioids

## Abstract

Drug–drug interactions (DDIs) are a leading cause of morbidity and mortality. New tools are needed to improve identification and treatment of DDIs. We conducted a randomized controlled trial to assess the clinical utility of a new test to identify DDIs and improve their management. Primary care physicians (PCPs) cared for simulated patients presenting with DDI symptoms from commonly prescribed medications and other ingestants. All physicians, in either control or one of two intervention groups, cared for six patients over two rounds of assessment. Intervention physicians were educated on the DDI test and given access to these test reports when caring for their patients in the second round. At baseline, we saw no significant differences in making the DDI diagnosis (*p* = 0.071) or DDI-related treatment (*p* = 0.640) between control and intervention arms. By round two, providers who accessed the DDI test performed significantly better in making the DDI diagnosis (+41.6%) and performing DDI-specific treatment (+12.2%) than in the previous round, and were 9.8 and 20.4 times more likely to diagnose and identify the DDI (*p* < 0.001 for all). The introduction of a definitive DDI test significantly increased identification, appropriate management, and counseling of DDIs among PCPs, which has the potential to improve clinical care.

## 1. Introduction

Drug–drug interactions (DDIs) are a strong driver of hospitalizations, emergency room visits, and a leading cause of morbidity and mortality [1,2,3]. Today, several factors threaten to increase the incidence rate of DDIs, including an aging patient population, concomitant use of multiple medications, and rising prescription abuse rates [4]. Despite the clinical urgency, providers are significantly challenged with identifying DDIs [5]. Patients may omit (intentionally or unintentionally) medications, supplements, or over-the-counter medications (OTC) as well as unknowingly ingest foods that may interfere with medications during reconciliation of home medication lists. These factors all contribute to the variability in the diagnosis of DDIs [6]. Even in the more controlled inpatient setting, complete with pharmacy decision support systems and medication reconciliation reports, DDIs account for 20% of all adverse drug events [7].

General awareness and the complexity of medical regimens has led to a couple of mainstays in modern practice that could potentially mitigate DDIs. Providers rely on clinical decision support systems to assist in identifying DDIs. However, the evidence suggests that reliance on these systems alone can lead to suboptimal identification of important DDIs and that providers become desensitized to alerts [8,9]. Pharmacy medication reconciliation reports, another practice standard, are also handicapped as medical records are often incomplete and insights regarding potential drug interactions are only as good as the data that is inputted [10].

Laboratory testing for DDIs has the potential to overcome these problems. Existing presumptive urine drug tests (e.g., qualitative immunoassays) lack the breadth and specificity to confirm the ingestion of many commonly used drugs, OTCs, and foods capable of causing DDIs. Even when definitive urine drug testing is used (e.g., liquid chromatography-mass spectrometry), physicians may not understand the severity and impact of the DDI to make appropriate changes to their patients’ medications [11]. A new DDI test (InterACT Rx^TM^) utilizes a sensitive and specific liquid chromatography mass spectrometry-based urine test to detect substances subject to DDIs and reference detected substances to a comprehensive database of DDIs for the ordering physician. The test results are delivered in a succinct report to providers along with actionable recommended changes to their patient’s drug regimens or treatment plans. The DDI Effectiveness and Clinical Awareness Randomized-Controlled Trial (DECART) was designed to experimentally determine if this technology improves diagnostic accuracy and appropriately alters clinical therapy for some of the most common DDI interactions.

## 2. Materials and Methods

DECART is a randomized, controlled cross-sectional study conducted between May and September 2018. The study examined the DDI-related preventive care practices of clinically-active primary care physicians (PCPs) in the U.S. We collected two rounds of clinical practice data on DDI identification with and without the new DDI test. A total of 313 PCPs from across the U.S. cared for a total of six simulated Clinical Performance and Value^®^ (CPV^®^) patients, three in each assessment round. With simulated patients, we obviated the one-off, variable nature of the patient presentation which allowed us to directly compare participant responses and examine whether better testing changed the workup, diagnosis, and care recommendations of providers [12].

### 2.1. Ethics

This study was conducted in accordance with ethical standards, approved by the Advarra Institutional Review Board, Columbia, MD, USA, and listed in clinicaltrials.gov (NCT03581994). Informed consent was obtained from all participants.

### 2.2. Physician Selection

We recruited participants from a nationally representative list of over 25,000 PCPs, compiled from relevant contact resources, including medical association workforce databases and list serves, hospital organization physician rosters, and national medical conference attendees. Between May and July 2018, we invited randomly selected physicians from the compiled list to participate in the DECART study.

Eligible participants had to (1) be physicians either board-certified in internal medicine or family medicine, (2) practice in a non-academic setting, (3) have between two and 30 years of post-residency practice, and (4) have an active panel of over 500 patients with an adult patient load of more than 50%. The final physician roster was randomized into three groups (control, intervention 1, and intervention 2) in a ratio of 1:1:1. In the first round of data collection, all providers were naïve to the DDI test and did not have access to any DDI test results. Prior to the second round, only the two intervention arms were given intervention educational materials. However, while Intervention 1 providers were all given the DDI test reports when caring for each of their CPV patients, Intervention 2 providers had the option to order (or not order) the DDI test when working up any of their CPV patients.

### 2.3. Intervention

The DDI test, a laboratory analysis of a urine specimen by mass spectrometry paired with clinically actionable information regarding identified DDIs, was introduced to intervention provider groups via physician-targeted educational materials. These materials, given after round one to those selected into either intervention group, provided overviews of the offering. The DDI test was referred to only as a ‘Definitive DDI Test’ and no brand name was used. The five educational materials provided were (1) a two-page marketing pamphlet of the DDI test, (2) a detailed, two-page document providing background on DDIs (prevalence, types, and current identification tools), (3) an 8-minute webinar overview of DDIs and the test offering, (4) an example test report, and (5) an example case study. We provided the materials to the intervention physicians approximately two to three weeks after completing the first round. Approximately two weeks after review of all educational materials, all intervention and control group physicians were asked to complete three additional CPV cases in the second round.

### 2.4. The Simulated Patients

CPVs are online simulated patients that a physician cares for just as they would in their physical clinical practice [13]. The open-ended vignette environment allows physicians to (1) request and review patient histories, (2) make a physical examination of the patient, and (3) order diagnostic tests and procedures to recreate an actual patient visit. Once participants complete these domains of care, they are then charged with (4) making a diagnosis with a treatment plan and follow-up. Two independent physician scorers, using explicit, pre-determined criteria, then evaluate each of the participating physician’s responses. A third physician adjudicates in the case of a disagreement on any of the individual criteria. Each domain, as well as the overall case, is given a percentage score between 0% and 100% depending on the number of evidence-based practice criteria completed. Previous studies have shown that a minimum 3–5% change in CPV scores is indicative of real behavioral change and subsequent improvement in patient outcomes [14].

### 2.5. CPV Cases

The CPV cases simulated DDIs involving one of three commonly prescribed medication classes (opioids, CNS depressants, and psychiatric medications) that occurred due to concomitantly ingested substances (OTCs/supplements/food, antimicrobials) or involved complex patients (alcohol use/polypharmacy) (Table S1).

### 2.6. Analysis

The primary outcome was to determine whether use of the DDI test demonstrated clinical utility and improved patient care through changes in medication management. More specifically, after controlling for provider and clinical practice characteristics, we sought to (1) determine if there was any improvement in diagnosing and identifying the specific DDIs and in treating or resolving the DDI after the physician received the DDI test reports and (2) explore how effective the intervention materials were in promoting DDI testing after being introduced to the new technology. For categorical dependent outcomes, we used either the chi-square test for single binary independent variables and logistic regression for multivariate modeling. Analogously for analyses involving continuous outcomes, t-tests and linear regression modeling were performed. All analyses were done in Stata 14.2. (StataCorp, LLC, College Station, TX, USA).

## 3. Results

The primary aim of the DECART study was to determine the clinical utility of a new liquid chromatography mass spectrometry urine test and its ability to assist practicing PCPs to identify, specify, and treat patients that are prone to adverse drug events secondary to a DDI.

We first compared the baseline provider and practice characteristics of the control and intervention arms among the 313 board-certified family or internal medicine physicians that met the eligibility criteria (Table 1 and Table S2). We found no significant differences between any of the study arms with the exception that participants in intervention 1 had a higher share of self-paying patients (7.1% vs. 4.4%; *p* = 0.005). With respect to diagnosis and treatment, prior to introducing the new DDI test, control physicians scored 2.6% points higher in the diagnosis + treatment domain than the intervention physicians (*p* = 0.046) (Table 2). Deeper exploration focusing on DDI-related treatment performance alone, revealed no statistical difference (*p* = 0.640) (Table 3).

After the introduction of the DDI test, the round two data showed a marked improvement in diagnosis + treatment in the intervention arms compared to the control group (Table 2 and Table 3). Intervention 1 improved by 5.5% (*p* < 0.001) round-over-round in the aggregate diagnosis + treatment scores, whereas controls regressed 4.3% (*p* = 0.001) over that same period for a net difference of 9.8%. This exceeds the clinically detectable 3–5% threshold that is needed to improve patient outcomes [14].When we looked at diagnostic accuracy alone, intervention 1 (who were all given the DDI test reports) demonstrated a 40.4% improvement in making the primary DDI diagnosis, while among the second intervention group (who had the option to order the DDI test) we observed a more modulated 13.4% improvement over the first round (*p* < 0.001 for both; Table 4). Both intervention groups had dramatic improvements in other specific DDI-related diagnosis and treatment items: identifying the potentially harmful DDI, advising the patient on the risks of these potential DDIs, and advising the patient to stop taking the interacting drug (*p* < 0.05 for all; Table 5, Table 6 and Table 7). This was in comparison to the control group who showed regression or nonsignificant improvement in DDI-related performance.

Restricting our view to the second intervention group (who had the option to order the DDI test), the DDI diagnostic test was ordered in 17.9% of cases. Among those who ordered the DDI test, these providers went on to diagnose the potential DDI significantly more often compared to those who did not order the new test (67.9% vs. 16.4%; *p* < 0.001). Interestingly, those who did not order the test were statistically indifferent from the controls (67.9% vs. 56.7%; *p* = 0.140). When we compared the physicians that ordered the new test in intervention 2 and the intervention 1 physicians who passively received the test reports, the physicians who actively ordered the new test trended better in most DDI-related diagnosis + treatment items but were only statistically better in discontinuing the interacting drug (78.3% vs. 60.9%, *p* = 0.030).

We symmetrically compared those in the second group who did not order the test with the control groups. This subset of physicians who did not choose to order the test, even after receiving the education materials, had similar DDI diagnosis rates as the controls (16.4% vs. 17.1%; *p* = 0.911) (Table 8).

To determine the covariates associated with the better diagnosis + treatment that were experimentally linked to advanced DDI testing, we performed multivariate regressions for all providers and cases, controlling for gender, age, specialty, region, practice locale and type, medication, and interacting drug, as well as study arm and round. We observed that there were no physician and practice characteristics associated with practice change in either the diagnosis + treatment performance or DDI-specific treatment. As in the bivariate analysis, providers who ordered the DDI test performed significantly better in ordering needed diagnosis-treatment (+9.5%) and introducing DDI-specific treatment (+12.2%) compared with those who did not order the test (*p* < 0.001 for both). By contrast, those who ordered the DDI test did not perform better in non-DDI-related treatment items (*p* = 0.459).

Overall, when we looked at specific DDI-related diagnosis and treatment, ordering the DDI test (whether as a passive recipient in intervention 1 or opting to order the DDI test in intervention 2) resulted in being 20.4 times more likely to identify the potentially harmful DDI (95% C.I. 4.2–99.1), 12.6 times more likely to advise patient on potential DDI risks (95% C.I. 3.9–41.1), and 15.1 times more likely to stop the interacting drug (95% C.I. 6.5–34.9) (Table 9).

Notably, in these regression analyses, we controlled for other types of urine tests (urinalysis, presumptive urine drug test, and definitive urine drug test). None of these other types of urine tests significantly increased the likelihood of correctly identifying or ordering any of the above-specified items (*p* > 0.05 for all), with one exception. Those that ordered the presumptive test were more likely to advise their patient of DDI risks (O.R. 2.0, 95% C.I. 1.1–3.5).

Among the various drug–drug interactions included in this study, we found that patients on opioids were significantly less likely to be diagnosed as having a DDI (O.R. 0.68, 95% C.I. 0.53–0.88) compared to patients on either psychiatric medications or CNS depressants, with subsequent reductions in having the DDI identified (O.R. 0.46, 95% C.I. 0.27–0.80) and having the interfering drug stopped (O.R. 0.30, 95% C.I. 0.23–0.40). However, patients on opioids were nearly as likely to be advised on the risks of a potential DDI as other patients (O.R. 0.91, 95% C.I. 0.61–1.35). Similarly, when we looked at the interfering drugs (OTC/supplements/food, antimicrobials, and alcohol/polypharmacy), patients with possible interactions of alcohol/polypharmacy were significantly less likely to be diagnosed with a DDI (O.R. 0.53, 95% C.I. 0.41–0.69), have the DDI identified (O.R. 0.29, 95% C.I. 0.15–0.55), be advised of DDI risks (O.R. 0.53, 95% C.I. 0.32–0.87), and have the interacting drug stopped (O.R. 0.24, 95% C.I. 0.17–0.34).

To examine the effectiveness of the new DDI test in identifying the diagnosis of specific DDIs, we restricted the regression analyses by medication class and found that ordering the DDI test increased the likelihood of diagnosing the potential for a harmful opioid interaction by 31.2 times (95% C.I. 6.5–149.4), by 9.6 times for psychiatric medications (95% C.I. 2.2–42.3), and by 41.4 times for CNS depressants (95% C.I. 6.1–279.4). Similarly, when we restricted the regression analyses by the interacting drug, ordering the DDI test increased the likelihood of a DDI diagnosis by 23.3 times for antimicrobial interactions (95% C.I. 3.8–143.2), 87.7 times for OTC/supplements/food interactions (95% C.I. 16.8–457.2), and 9.0 times for polypharmacy/alcohol interactions (95% C.I. 1.9–42.7).

## 4. Discussion

There is significant opportunity to improve physicians′ awareness of DDIs and prevent complications and even deaths. Two separate national surveys [11,15] found providers identified DDIs in less than half of cases. Our findings in *Part 1* of the DECART study confirm this and suggest that the problem could be far worse given less than one-fifth of participants identified DDIs in their CPV-simulated patient cases [6]. We believe our estimates may be more representative of what is seen in real practice because CPVs simulate everyday patient encounters wherein providers prescribe treatment recommendations in the context of a clinical setting, as opposed to directed questions on specific DDIs outlined in traditional surveys. The magnitude of these observations is underscored by the observation that in the aforementioned studies, providers had the opportunity to reference external resources and look up drug interactions online, via a drug database. These low DDI detection findings suggest referencing DDI resources may not be in the regular routine of provider practice.

Our findings in *Part 2* of the DECART study showed that the introduction of a definitive DDI test improved DDI identification nearly 10-fold. Not only did identification improve, but subsequent, appropriate DDI care did as well: providers that used the DDI test were over 15 times more likely to make changes to the patient’s regimen to stop the DDI from occurring.

This change in practice has the potential to produce significant benefits to the patient and savings to the healthcare system. Patients exposed to a DDI have been shown to experience significantly more office visits, outpatient visits, emergency department visits, inpatient hospitalizations, and longer inpatient length of stay [16]. Taylor et al. found that, in non-cancer pain patients, median 6-month expenditures were as much as $1070 more per patient with a DDI compared to a patient without. Providers who used the DDI test were 12.6 times more likely to counsel patients on DDIs and their risks. This increase in counseling is advantageous not only for the treatment and avoidance of known DDIs but also for possible prevention of others. One study found that that there was ample room to improve patient awareness of DDIs, as well as vigilance for potential interactions of all drugs, including those sold over the counter [17]. This study confirmed there was not only opportunity to improve awareness but that counselling was associated with better management. Other researchers could help to quantify the impact of DDI education and awareness on providing more complete medication histories and any additional substances they are taking.

The two intervention arms in this study provided insights on the challenges of getting providers to improve their practice. We observed a large disparity between those who were automatically delivered test reports versus those who received test reports only if they chose to order the DDI test (17.9%) suggesting that there is a significant knowledge gap that we did not adequately bridge in this study. The magnitude of this difference was surprising, as both intervention groups had access to identical educational materials. Perhaps this is a reflection of the educational materials selected, but we and others feel that DDIs remain overlooked and, as such, do not generate enough clinical urgency to alter therapy [5]. *Part 1* of the DECART study further supports this. The upfront survey conducted herein indicates that there might also be an unwarranted sense of confidence that DDIs are caught with their current DDI detection practices, where 88% and 75% indicated they relied on pharmacy or computerized detection tools, respectively [6]. These disparities and the rising tendency for physicians to override system alerts [18] suggests these tools are not working.

With the enormous pressure primary care providers face to see a rising number of patients and provide comprehensive care, it is small wonder that critical findings are missed [19]. As seen in the results of this study, the utilization of a novel test that allows for identification of DDIs between definitively identified ingested substances significantly improved providers’ abilities to diagnosis interactions and appropriately treat patients. Further, with an aging demographic, increases in polypharmacy regimens, and rising drug abuse there may be a role for routine DDI testing in at-risk patients, for example patients with memory loss, a history of substance abuse, therapeutic futility, or questions of compliance. Future studies will have to explore our findings of underdiagnosis and inadequate treatment.

Our study has several limitations. Although efforts were made to match demographics of practicing PCPs and patients in the U.S., our final participant population had a higher representation of men and middle age physicians, and participating physicians reported having slightly fewer patients who consumed alcohol (55.8% vs. 73%) or were on opioid analgesics (13.9% vs. 38%) than what is expected in the general population. This difference could be due to a prevalent underreporting of opioid prescribing generally seen [20]. Another shortcoming is that, while we studied some of the most common DDIs ranging from antibiotics to analgesics to CNS depressants with alcohol and opioids, there are a large number of DDIs that exist and are potentially even more under-recognized than the ones included in this study. Additionally, we did not include patient-level data into our design. CPV studies have already been validated as measuring actual clinical practice [13] and established as an innovative way to determine clinical utility [12].

This study clearly shows that there is a need to improve physician identification of DDIs and that new definitive DDI tests, like InterACT Rx, could help to do so. Secondly, this study demonstrates that definitive identification of commonly prescribed medications and other ingestants results in increased identification of DDIs, improvement in medication management, and enhances patient outcomes.

## Figures and Tables

**Table 1:** Provider Characteristics.

Variables	Overall (313)	Control (109)	Intervention 1 (100)	Intervention 2 (104)	*p*-Value
**Male**	76.7%	81.7%	75.0%	73.1%	0.296
**Age**					
<40	6.1%	8.3%	7.0%	2.9%	0.119
40–55	60.4%	53.2%	67.0%	61.5%	
>55	33.6%	38.5%	26.0%	35.6%	
**Board certification**					
Family medicine	48.6%	52.3%	50.0%	43.3%	0.718
Internal medicine	50.2%	46.8%	49.0%	54.8%	
Both	1.3%	0.9%	1.0%	1.9%	
**Years in practice**	20.2 ± 6.9	20.3 ± 7.6	19.7 ± 6.8	20.7 ± 6.3	0.581
**Active panel size**	2561 ± 1529	2474 ± 1429	2571 ± 1476	2642 ± 1683	0.723
**Patient panel characteristics**					
On 5 or more medications	41.3% ± 21.7%	41.1% ± 22.1%	41.7% ± 22.0%	41.3% ± 21.1%	0.980
On opioid analgesics	13.8% ± 10.8%	13.4% ± 10.7%	13.7% ± 9.8%	14.3% ± 11.9%	0.849
≥2 alcoholic beverages/month	56.1% ± 23.6%	58.3% ± 23.6%	55.4% ± 22.8%	54.3% ± 24.4%	0.436
**Payer type**					
Medicare	34.2% ± 14.4%	35.8% ± 14.3%	34.6% ± 14.8%	32.0% ± 14.1%	0.145
Medicaid	10.6% ± 11.8%	8.9% ± 9.1%	11.9% ± 13.7%	11.3% ± 12.0%	0.142
Commercial	48.4% ± 18.2%	49.2% ± 17.8%	45.1% ± 17.8%	50.9% ± 18.6%	0.063
Self	5.3% ± 6.8%	4.1% ± 4.9%	7.1% ± 9.3%	4.8% ± 5.3%	0.005
Other	1.5% ± 3.9%	2.0% ± 5.3%	1.4% ± 3.0%	1.1% ± 2.6%	0.221

**Table 2:** DDI-related performance by intervention arm and round: Diagnosis-treatment domain performance.

	Control	Intervention 1	Intervention 2	*p*-Value
Round 1	24.7% ± 17.4%	22.7% ± 16.8%	21.4% ± 16.3%	0.046
Round 2	20.4% ± 16.0%	28.2% ± 16.5%	21.7% ± 16.9%	<0.001
*p*-value	0.001	<0.001	0.798	

**Table 3:** DDI-related performance by intervention arm and round: DDI-related treatment performance.

	Control	Intervention 1	Intervention 2	*p*-Value
Round 1	24.6% ± 25.5%	23.1% ± 24.6%	22.9% ± 24.8%	0.640
Round 2	15.7% ± 19.4%	27.7% ± 24.1%	18.1% ± 20.8%	<0.001
*p*-value	<0.001	0.021	0.009	

**Table 4:** DDI-related performance by intervention arm and round: Diagnosis of DDI.

	Control	Intervention 1	Intervention 2	*p*-Value
Round 1	18.7%	16.3%	12.2%	0.071
Round 2	17.1%	56.7%	25.6%	<0.001
*p*-value	0.611	<0.001	<0.001	

**Table 5:** DDI-related performance by intervention arm and round: Identification of specific DDI.

	Control	Intervention 1	Intervention 2	*p*-Value
Round 1	1.5%	0.7%	0.6%	0.529
Round 2	4.0%	18.7%	3.9%	<0.001
*p*-value	0.092	<0.001	0.012	

**Table 6:** DDI-related performance by intervention arm and round: Advise patient of potential DDI.

	Control	Intervention 1	Intervention 2	*p*-Value
Round 1	8.7%	6.1%	2.8%	0.008
Round 2	2.1%	21.4%	7.5%	<0.001
*p*-value	<0.001	<0.001	0.018	

**Table 7:** DDI-related performance by intervention arm and round: Advise patient to stop interacting drug.

	Control	Intervention 1	Intervention 2	*p*-Value
Round 1	20.3%	21.3%	21.0%	0.965
Round 2	22.6%	60.9%	30.6%	<0.001
*p*-value	0.543	<0.001	0.011	

**Table 8:** Round 2 Comparison Results of: (a) Intervention 2 non-DDI test orderers vs. Controls and (b) Intervention 2 DDI test orderers vs. Intervention 1.

Variables	Control (327)	Intervention 2, Did Not Order DDI (256)	*p*-Value	Intervention 1 (300)	Intervention 2, Ordered DDI (56)	*p*-Value
**Domain**						
History	62.8 ± 13.7	61.4 ± 14.2	0.237	65.6 ± 14.3	68.9 ± 12.7	0.102
Physical	83.8 ± 18.5	81.6 ± 18.7	0.153	84.6 ± 17.7	92.0 ± 14.2	0.003
Workup	42.5 ± 39.1	38.9 ± 35.4	0.251	90.7 ± 16.9	91.7 ± 16.0	0.691
Diagnosis + Treatment	20.4 ± 16.0	19.5 ± 16.4	0.488	28.2 ± 16.5	31.9 ± 15.7	0.117
**Treatment**						
DDI-related	15.7 ± 19.4	15.0 ± 19.7	0.672	27.7 ± 24.1	32.1 ± 20.4	0.203
Non-DDI-related	16.6 ± 23.2	15.8 ± 23.6	0.700	16.8 ± 22.7	20.9 ± 25.4	0.226
**Individual Items**						
Workup						
CMP	92.7%	97.5%	0.100	91.7%	100.0%	0.366
CBC	94.9%	100.0%	0.499	86.1%	100.0%	1.000
Coagulation	24.3%	32.0%	0.569	54.1%	33.3%	0.412
ECG	53.4%	56.3%	0.777	52.4%	43.8%	0.597
Urinalysis	30.6%	34.8%	0.286	34.7%	30.4%	0.645
Presumptive Urine Drug Test	44.7%	35.2%	0.022	11.3%	44.6%	<0.001
Definitive Urine Drug Test	17.7%	13.7%	0.210	8.0%	25.0%	0.001
Primary DX						
DDI diagnosed	17.1%	16.4%	0.911	56.7%	67.9%	0.140
DDI identified	4.0%	1.2%	0.043	18.7%	16.1%	0.711
DDI Treatment Items						
Advise DDI	2.1%	3.6%	0.413	21.4%	26.1%	0.564
Advise other	7.8%	7.1%	1.000	15.3%	23.1%	0.385
Stop meds	22.6%	20.7%	0.666	60.9%	78.3%	0.030
Change meds	31.3%	28.0%	0.442	30.0%	34.6%	0.515

**Table 9:** DDI-Specific Logistic Regression Results of Different Urine Tests ^†^.

	Diagnose Potentially Harmful DDI			Identify Potentially Harmful DDI			Advise Patient on DDI Risks and Effects			Order Stop of Interacting Drug		
	OR	95% Lower	95% Upper	OR	95% Lower	95% Upper	OR	95% Lower	95% Upper	OR	95% Lower	95% Upper
Urinalysis	1.2	0.9	1.6	1.4	0.9	2.3	0.9	0.6	1.4	1.2	1.0	1.6
Presumptive urine drug test	0.9	0.6	1.2	0.6	0.3	1.2	2.0 *	1.1	3.5	1.0	0.7	1.4
Definitive urine drug test	1.1	0.8	1.6	0.9	0.3	2.4	0.7	0.3	1.3	0.9	0.6	1.3
DDI diagnostic test	9.8 **	4.8	20.0	20.4 **	4.2	99.1	12.6 **	3.9	41.1	15.1 **	6.5	34.9

^†^ Regression results controlled for provider characteristics (gender, age, specialty, region, practice locale and type), medication, and interacting drug, as well as study arm and round. * *p*-value < 0.05; ** *p*-value < 0.001.

## Supplementary Materials

The following are available online at http://www.mdpi.com/2077-0383/7/11/384/s1, Table S1: List of Substances and Markers Tested by Definitive Urine Drug Test and DDI, Table S2: Continued List of Provider Characteristics.
