# Supplementary material for: Clinical Utility of Definitive Drug–Drug Interaction Testing in Primary Care
Source: J Clin Med. 2018 Oct 25;7(11):384. doi: 10.3390/jcm7110384 (PMC6262337; doi:10.3390/jcm7110384)
Supplement: Supplementary file 1 [file jcm-07-00384-s001.pdf]

Supplemental Table 1. List of Substances and Markers Tested by Definitive Urine Drug Test and DDI Test.

**Substances Included in Definitive Urine Drug Test**	**Markers Tested for Ingestion of Substances**
Alprazolam	Alprazolam, alpha-hydroxy-Alprazolam
Amphetamine	Amphetamine
Buprenorphine	Buprenorphine, Norbuprenorphine
Carisoprodol/Meprobamate	Carisoprodol, Meprobamate
Chlordiazepoxide	Nordiazepam, Oxazepam
Clonazepam	7-aminoclonazepam
Clorazepate	Nordiazepam, Oxazepam
Cocaine	Cocaine, Benzoylecognine
Codeine	Codeine, Norcodeine, Morphine, Hydrocodone (Minor)
Diazepam	Nordiazepam, Temazepam, Oxazepam
Dihydrocodeine	Dihydrocodeine
Ethyl Glucuronide/Ethyl Sulfate	Ethyl Glucuronide, Ethyl Sulfate
Fentanyl	Fentanyl, Norfentanyl
Flurazepam	2-hydroxyethyl-flurazepam
Gabapentin	Gabapentin
Heroin	Heroin, 6-monoacetylmorphine, 6-acetylcodeine, Morphine, Codeine
Hydrocodone	Hydrocodone, Norhydrocodone, Hydromorphone, Dihydrocodeine
Hydromorphone	Hydromorphone
Lorazepam	Lorazepam
MDA	MDA
MDEA	MDEA, MDA
MDMA	MDMA, MDA
Meperidine	Meperidine, Normeperidine
Methadone	Methadone, EDDP
Methamphetamine	Methamphetamine, Amphetamine
Morphine	Morphine, Hydromorphone (Minor)
Nicotine	Cotinine, 3-hydroxycotinine
Oxazepam	Oxazepam
Oxycodone	Oxycodone, Noroxycodone, Oxymorphone
Oxymorphone	Oxymorphone
Phentermine	Phentermine
Pregabalin	Pregabalin
Tapentadol	Tapentadol, Nortapentadol
Temazepam	Temazepam, Oxazepam
Tramadol	Tramadol, N-desmethyltramadol, O-desmethyltramadol
Substances included in DDI Test (in addition to above)	Markers Tested for Ingestion of Substances
Abiraterone	Abiraterone
Acyclovir	Acyclovir
Albuterol	Albuterol
Amiodarone	Amiodarone, Desethylamiodarone
Amitriptyline	Amitriptyline, Nortriptyline
Amlodipine	Amlodipine, Amlodipine Metabolite
Apixaban	Apixaban
Asenapine	Asenapine
Atazanavir	Atazanavir
Atomoxetine	Atomoxetine, 4-OH-Atomoxetine
Atorvastatin	Atorvastatin, Atorvastatin Lactone
Avanafil	Avanafil
Azithromycin	Azithromycin, N-desmethyl Azithromycin
Bergamottin (Grapefruit Furanocoumarins)	Bergaptol, Dihydroxybergamottin
Bupropion	Bupropion, Hydroxy Bupropion
Butalbital	Butalbital
Canagliflozin	Canagliflozin
Carbamazepine	Carbamazepine Epoxide

Chloramphenicol	Chloramphenicol
Chloroquine	Chloroquine
Chlorpromazine	Chlorpromazine
Cimetidine	Cimetidine
Ciprofloxacin	Ciprofloxacin
Citalopram/Escitalopram	Citalopram/Escitalopram, N-desmethylcitalopram/N-desmethylescitalopram
Clarithromycin	Clarithromycin
Clobazam	Clobazam, Desmethylclobazam
Clomipramine	Clomipramine
Clopidogrel	Clopidogrel, Clopidogrel Carboxylate
Cobicistat	Cobicistat
Cyclobenzaprine	Cyclobenzaprine, Norcyclobenzaprine
Darunavir	Darunavir
Delavirdine	Delavirdine
Desipramine	Desipramine
Desvenlafaxine	O-desmethylvenlafaxine
Dexamethasone	Dexamethasone
Diltiazem	Diltiazem, Deacetyl diltiazem n-oxide
Donepezil	Donepezil
Doxepin	Doxepin, Nordoxepin
Doxorubicin	Doxorubicin
Duloxetine	Duloxetine
Efavirenz	Efavirenz, 8-hydroxyefavirenz
Enzalutamide	Enzalutamide
Erythromycin	Erythromycin, N-desmethyl Erythromycin A
Ethinyl Estradiol	Ethinyl Estradiol, 2-Methoxy Ethinyl Estradiol
Etravirine	Etravirine
Famotidine	Famotidine
Fluconazole	Fluconazole
Fluoxetine	Fluoxetine, Norfluoxetine
Fluphenazine	Fluphenazine
Fluvoxamine	Fluvoxamine, Fluvoxamine Acid
Formoterol	Formoterol
Fosamprenavir	Fosamprenavir, Amprenavir
Guanfacine	Guanfacine
Haloperidol	Haloperidol
Iloperidone	Iloperidone
Indinavir	Indinavir
Itraconazole	Itraconazole, 2-Hydroxyitraconazole
Kava	Dihydrokavain
Ketoconazole	Ketoconazole
Lansoprazole	Lansoprazole, OH-Lansoprazole Sulfide, Lansoprazole N-Oxide
Levofloxacin/Ofloxacin	Levofloxacin/Ofloxacin
Lorcaserin	Lorcaserin
Medroxyprogesterone Acetate	Medroxyprogesterone Acetate, Medroxyprogesterone
Methadone	Methadone, EDDP
Methylprednisolone	Methylprednisolone
Metoclopramide	Metoclopramide
Metronidazole	Metronidazole, OH-Metronidazole
Mirabegron	Mirabegron
Nefazodone	Nefazodone
Nelfinavir	Nelfinavir, 2-Hydroxynelfinavir
Nevirapine	Nevirapine
Nifedipine	Nifedipine, Nifedipine Carboxylate
Nilotinib	Nilotinib
Nortriptyline	Nortriptyline
Omeprazole/Esomeprazole	Omeprazole/Esomeprazole, 5-OH Omeprazole, Omeprazole Sulfone, 5-O-Desmethyl Omeprazole, 4-OH-Omeprazole Sulfide

Ondansetron	Ondansetron
Oxcarbazepine	Oxcarbazepine
Paroxetine	Paroxetine, Paroxetine Metabolite I, Paroxetine Metabolite II, Paroxetine Metabolite III
Pazopanib	Pazopanib
Perphenazine	Perphenazine
Phenobarbital	Phenobarbital
Phenytoin	Phenytoin
Pioglitazone	Pioglitazone, Hydroxypioglitazone
Posaconazole	Posaconazole
Pramipexole	Pramipexole
Prednisone	Prednisone
Primidone	Primidone, Phenobarbital
Promethazine	Promethazine, Promethazine Sulfoxide
Propranolol	Propranolol
Quetiapine	Quetiapine, Norquetiapine, 7-OH-Quetiapine
Quinidine	Quinidine
Quinine	Quinine
Ranitidine	Ranitidine, Desmethylranitidine
Ranolazine	Ranolazine
Rifabutin	Rifabutin
Rifampin	Rifampin
Rifapentine	Rifapentine
Risperidone	Risperidone
Ritonavir	Ritonavir, Hydroxy Ritonavir
Rivaroxaban	Rivaroxaban
Ropinirole	Ropinirole, N-Despropyl Ropinirole
Salmeterol	Salmeterol
Saquinavir	Saquinavir
Sertraline	Sertraline, Norsertraline
St. John's wort	Hyperforin
Sumatriptan	Sumatriptan, Sumatriptan Metabolite
Thioridazine	Thioridazine
Ticlopidine	Ticlopidine
Tipranavir	Tipranavir
Tizanidine	Tizanidine, Dehydrotizanidine
Trazodone	Trazodone
Valacyclovir	Valacyclovir
Vemurafenib	Vemurafenib
Venlafaxine	Venlafaxine, O-desmethylvenlafaxine
Verapamil	Verapamil, Norverapamil
Voriconazole	Voriconazole, Voriconazole N-Oxide
Warfarin	Warfarin, 7-OH-Warfarin
Zileuton	Zileuton

Supplemental Table 2. Continued List of Provider Characteristics.

Variables	Overall (313)	Control (109)	Intervention 1 (100)	Intervention 2 (104)	*p*-value
**Fellowship**	14.4%	11.0%	19.0%	13.5%	0.260
**Years in practice**	20.2 + 6.9	20.3 + 7.6	19.7 + 6.8	20.7 + 6.3	0.581
**Region**					
Midwest	22.4%	23.9%	21.0%	22.1%	0.547
Northeast	26.5%	21.1%	27.0%	31.7%	
South	31.3%	30.3%	33.0%	30.8%	
West	19.8%	24.8%	19.0%	15.4%	
**Locale**					
Urban	26.5%	31.2%	20.0%	27.9%	0.161
Suburban	61.3%	59.6%	62.0%	62.5%	
Rural	12.1%	9.2%	18.0%	9.6%	
**Practice type**					
Solo private	22.7%	21.1%	29.0%	18.3%	0.584
Single specialty private	38.7%	38.5%	34.0%	43.3%	
Multi-specialty private	32.9%	33.9%	33.0%	31.7%	
Hospital	5.8%	6.4%	4.0%	6.7%	
**Employed by practice, %**	74.8%	71.6%	71.0%	81.7%	0.132
**Multi-specialty practice**	32.6%	31.2%	35.0%	31.7%	0.846
**Medical practice setting (can choose more than one)**					
Accountable care organization	24.9%	31.2%	21.0%	22.1%	0.179
Solo practice	22.7%	21.1%	28.0%	19.2%	0.301
Group practice	67.4%	69.7%	62.0%	70.2%	0.393
Hospital-based	8.0%	7.3%	6.0%	10.6%	0.493
Integrated delivery system	11.8%	11.9%	11.0%	12.5%	0.975
Network model HMO	1.3%	2.8%	0.0%	1.0%	0.330
Staff-model HMO (employed)	1.3%	2.8%	0.0%	1.0%	0.330
Staff-model HMO (contracted)	1.3%	2.8%	1.0%	0.0%	0.274
Other	1.9%	92.0%	3.0%	1.9%	0.453
**Type of medication reconciliation used (can choose more than one)**					
Pharmacy/medication reconciliation	88.2%	87.2%	86.0%	91.4%	0.437
Presumptive urine drug test	57.2%	55.1%	58.0%	58.7%	0.861
Definitive urine drug test	37.1%	33.9%	42.0%	35.6%	0.454
Digital pills	1.9%	1.8%	2.0%	1.9%	1.000
Self-report	62.6%	56.0%	68.0%	64.4%	0.183
EMR/automated software	75.1%	73.4%	78.0%	74.0%	0.735
None	1.3%	1.8%	0.0%	1.9%	0.552
**Time since last CME in pain management**					
≤12 months	41.9%	44.0%	37.0%	44.2%	0.322
13-24 months	16.1%	11.0%	19.0%	20.2%	
25-36 months	10.2%	10.1%	11.0%	9.6%	

>36 months	11.5%	14.7%	14.0%	5.8%	
Never taken	19.8%	20.2%	19.0%	20.2%	
**Receive quality bonus**	58.8%	58.7%	55.0%	62.5%	0.544
If yes, preventive measures metrics	89.1%	90.5%	83.6%	92.3%	0.306
